# A diagnostic dilemma: distinguishing a sulfasalazine induced DRESS hypersensitivity syndrome from a CD30 + lymphoma in a young patient

**DOI:** 10.1186/s12245-024-00665-7

**Published:** 2024-07-18

**Authors:** Natalija Aleksandrova, Jonas De Rop, Frederic Camu, Ives Hubloue, Katleen Devue

**Affiliations:** 1 Department of Emergency Medicine, ASZ Aalst, Aalst, Belgium; 2 Department of Endocrinology, ASZ Aalst, Aalst, Belgium; 3 Department of Endocrinology, UZ Brussel, Jette, Belgium; 4 Department of Emergency Medicine, UZ Brussel, Jette, Belgium

**Keywords:** Dress syndrome, Drug hypersensitivity, Sulfasalazine, RegiSCAR, CD30 + lymphoma

## Abstract

Drug reaction with eosinophilia and systemic symptoms (DRESS) is a severe hypersensitivity reaction characterized by cutaneous rash, lymphadenopathy, fever, eosinophilia, leukocytosis, and life-threatening organ dysfunctions. We describe the case of a 26 year old patient admitted to the Emergency Department for DRESS syndrome after sulfasalazine treatment for rheumatoid arthritis in the right knee. Whole body computer tomography showed multiple neck, chest, and abdominal lymphadenopathy with splenomegaly, massive ascites and severe hepatic cytolysis. Serology results for Epstein-Barr Virus (EBV), influenza, measles, rubella, hepatitis A and B were negative. The histologic analysis of skin, lymph node and bone marrow biopsies could not indicate a classical Hodgkin’s Disease or iatrogenic immunodeficiency/EBV-associated lymphoproliferative disorder (LPD), Hodgkin type. The relatively small caliber of the CD30 + immunoreactive blastoid cells in the lymph nodes suggested reactive immunoblasts rather than Hodgkin cells. The morphologic aspects of the lymph node biopsies with predominance of T-cells were compatible with the diagnosis of a sulfasalazine-induced DRESS syndrome as the patient had a high RegiSCAR score for DRESS. [DRESS Syndrome Foundation: Diagnosis and Treatment. (2023)] The patient’s complex clinical course, marked by two hospital admissions, highlights the challenges in diagnosing and managing DRESS. This case underscores the need for individualized care, close patient monitoring, and further research to better understand DRESS’s underlying mechanisms and optimal therapeutic strategies.

## Introduction

Many pharmacologic agents may trigger dermatologic eruptions based on immunologic (allergies) or non-immunologic mechanisms, as manifestations of immediate or delayed hypersensitivity reactions. Drug reaction with eosinophilia and systemic symptoms (DRESS) syndrome is a life-threatening disease with cutaneous presentation and internal organ involvement. Its mortality rate is about 10% [2]. The estimated overall population risk of DRESS varies between 1:1000 and 1:10,000 drug exposures [3–5].

Diagnosis is challenging due to the lack of experience with the condition, low incidence and variations in clinical presentation [6]. DRESS syndrome is characterized by a long latency period of 3–8 weeks which makes diagnosis further challenging by the fact that the causative medication often has been discontinued and needs to be retrospectively linked to the clinical presentation [7].

We report here the case of sulfasalazine-induced DRESS with histologic features of CD30 + lymphoma.

## Case report

A 26-year-old male Caucasian patient was admitted to the Emergency Department in Aalst, Belgium for persistent red confluent facial and upper thoracic skin rash, sore throat, nausea, palpitations, fever and general discomfort in the last one week. Clinical examination showed fever at 38.7 °C, tachycardia (130 bpm) with cardiac systolic murmur, pain in the left hypochondrium, and multiple painful lymphadenopathies in the head and neck regions. The rash originated from the neck area, spreading to the face, and later extending to the back and breast regions. The arms, legs, and abdomen exhibited mild symptoms. The rash appeared as red in color, confluent, blanching, macular, and accompanied by itching.

His medical history revealed intermittent monoarthritic inflammation and hydrops of the right knee in the last 2 years, with a new exacerbation of synovitis, high sedimentation rate (65 mm) and C-reactive protein (CRP) value (92 mg/L) with limited rise of rheumatoid factor (RF; 14 U/ml) in blood one month prior. ANA-immunofluorescence and ANCA determinations were negative. Arthrocentesis results showed significantly elevated white blood cell count (14,118/mm^3^). Rheumatoid arthritis or spondyloarthropathy was suspected and treatment with sulfasalazine (500 mg 5 times per day) started. The patient stopped this treatment himself after 23 days because of the onset of fever and rash. The patient’s previous medical history was relatively unremarkable, there were no chronic diseases, recurrent infections, or allergies reported. He did not take any chronic medications but occasionally used non-steroidal anti-inflammatory drugs.

Laboratory investigations disclosed mild anemia (hemoglobin 11.6 g/dL), significant non-cholestatic hepatic pathology (AST 78 U/L, ALT 224 U/L, LDH 533 U/L, GGT 132 U/L, direct bilirubin 0.8 mg/dL, albumin 29.4 g/L, alkaline phosphatase 145 U/L), increased ferritin level (1325 µg/L), disturbed coagulation profile (INR 1.4), high CRP value (78 mg/L), mild leukocytosis (WBC 10,300/mm³) with eosinophilia 4.5%, immunity for EBV, influenza, measles, rubella, hepatitis A and B. Other possible causes of acute hepatitis, such as hepatitis C and E, HIV, and paracetamol intoxication were excluded. Group A Streptococci tests to exclude potential erysipelas were negative. Echocardiography showed no arguments for endocarditis, but some pericardial effusion was detected. Abdominal echography found no abnormalities.

A presumptive diagnosis of delayed onset sulfasalazine-induced toxicity was based on the presence of the skin rash and liver dysfunction, with differential diagnosis of Kawasaki disease (presence of large lymph nodes in the neck), oncological lymphoproliferative disorders like Hodgkin and adult Still’s disease (fever, joint pain and rash). Hypereosinophilic syndrome and other differentials, such as chlamydia, syphilis, borrelia and adenovirus were also sought and excluded.

Patient was positive on all 7 criteria of RegiSCAR scoring system, 6 out of 7 on Japanese consensus group (HHV-6 reactivation was not tested) and all three criteria on Bocquet, Bagot, and Roujeau (1996) (Table 1) [8].

**Table 1:** DRESS Diagnostic Scoring systems (“DRESS Syndrome Foundation: diagnosis and treatment,” [1], Bocquet, Bagot, and Roujeau, [8])

RegiSCAR study group	Japanese consensus group	Bocquet et al.
More than 3 of the criteria are required for the diagnosis of DRESS: 1. Hospitalization 2. Reaction suspected to be drug related 3. Acute rash 4. Fever above 38 °C 5. Enlarged lymph nodes involving at least two sites 6. Involvement of at least one internal organ 7. Blood count abnormalities: Lymphocytes above or below laboratory limits, Eosinophils above laboratory limits (in percentage or absolute count), Platelets below laboratory limits	Typical DRESS (presence of all 7 criteria); atypical DIHS (all criteria present except lymphadenopathy and HHV-6 reactivation): 1. HHV-6 reactivation 2. Prolonged clinical symptoms 2 weeks after discontinuation of causative drug 3. Maculopapular rash developing > 3 weeks after starting drug 4. Fever above 38 °C 5. Lymphadenopathy 6. ALT > 100 U/L or other organ involvement 7. Leukocyte abnormalities (at least one): Leukocytosis (> 11 × 10^9^/L), Atypical lymphocytosis (> 5%), Eosinophilia (1.5 × 10^9^/L)	DRESS is confirmed by presence of 3 criteria: 1. Cutaneous drug eruption 2. Adenopathies > 2 cm in diameter or hepatitis (liver transaminases > 2 times upper limit of normal) (or) interstitial nephritis (or) interstitial pneumonitis (or) carditis 3. Hematologic abnormalities eosinophilia > 1.5 × 10^9^/L (or) atypical lymphocytes

Initial treatment was started with supportive saline infusion and antipyretic doses of paracetamol (acetaminophen; up to 2 g/day for fever > 38.5 °C). All non-steroidal anti-inflammatory medications, aspirin and sulfasalazine were withheld. The patient was transferred to the Gastroenterology Department because of the hepatic cytolysis pathology.

In the meantime, the skin rash increased with significant swelling of the face, pharynx and neck, dyspnea, coughing and hypoxia, requiring admittance to the intensive care unit the next day. Leukocytosis increased to 17,100/mm³ with eosinophilia 11% (1830/mm³). Hepatosplenomegaly and ascites developed, and pyrexia peaked at 39 °C. Several blood and sputum cultures gave no indication of an infectious agent.

Computer Tomography (CT)-scan of thorax and abdomen showed multiple bilateral prominent lymphadenopathies in the mediastinum, the pulmonary hilum and the axillary regions. Large lymph nodes were also found along the truncus coeliacus and the right common iliac artery. Hepatosplenomegaly, right pleural effusion and massive ascites were confirmed. FDG-PET CT-scan revealed several areas of metabolically highly active metabolic areas in the head and neck and axillary regions.

The patient was further treated with corticosteroids (methylprednisolone 40 mg daily or 0.5 mg/kg/day), adequate antipyretic coverage and correction of corticosteroid–induced hyperglycemic disturbance. Following clinical improvement, the patient was transferred to the department of Oncology-Hematology two days later for further diagnosis.

**Fig. 1:**
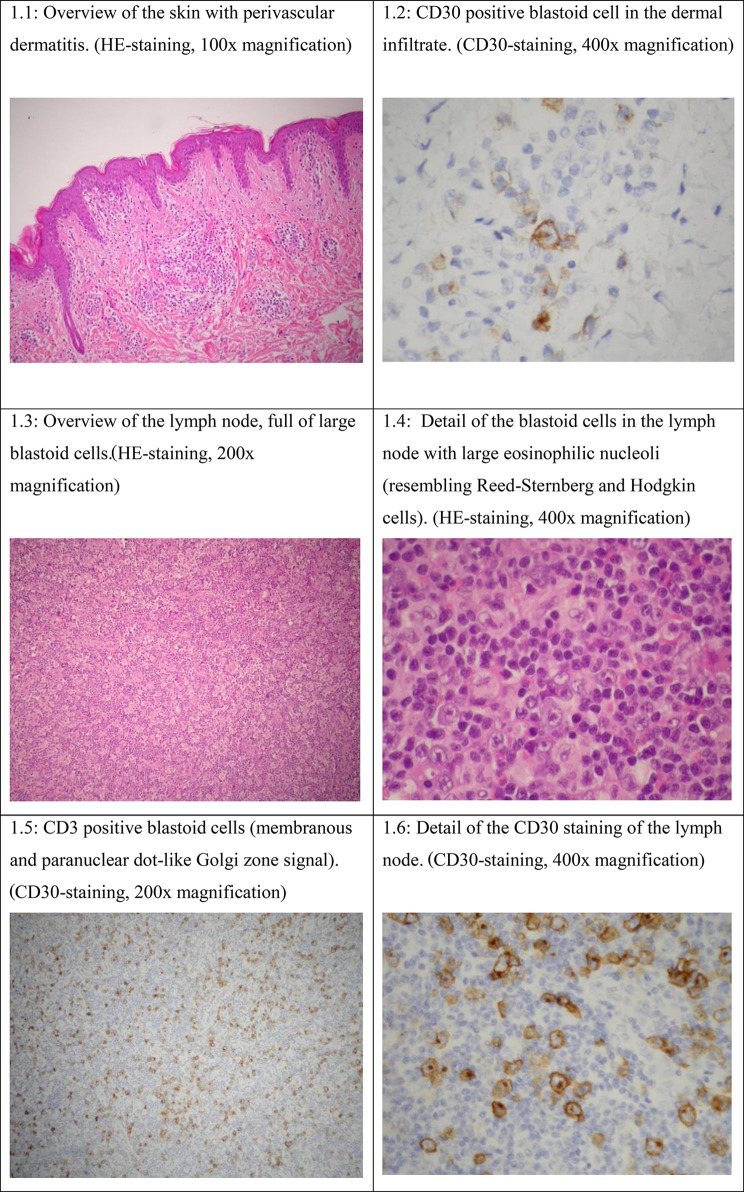
Biopsy results

Several biopsies were taken (skin, lymph node, bone marrow, Fig. 1).

The pathological anatomy of the skin biopsy revealed perivascular inflammation with mainly lymphocytes infiltration (Fig. 1.1) and the focal presence of large, atypical lymphocytes CD30 positive (Fig. 1.2). No eosinophilic granulocytes were seen.

The identification of CD30 + staining, in conjunction with the findings from tomography and clinical manifestations, has significantly increased our suspicion of lymphoma as a potential candidate in the differential diagnosis. The skin biopsy staining for CD15 + was not conducted. Immunohistochemical analysis showed no pathological IgG, IgA, IgM or complement deposits.

Two lymph nodes excision biopsies showed relatively preserved lymph node structure. There is a dominance of blastoid elements on background of small mature lymphocytes and increased number of eosinophilic granulocytes. There are several blastoid cell with large eosinophilic nucleoli resembling Reed-Sternberg and Hodgkin cells (Fig. 1.3, 1.4). Interestingly, Husain et al. (2013) concluded that Reed-Sternberg cells are absent in reported cases of DRESS, while they were present in a study by Stephan et al. (2016) [9, 10]. No immunoreactivity for CD3, CD5, CD15, CD20 or CD79α was found in the large, atypical cells while the smaller cells were almost exclusively CD3 and CD5 immunoreactive T-lymphocytes (Fig. 1.5). In situ hybridization for Epstein Barr virus was negative.

Given that in situ hybridization for EBV was negative, an EBV-associated LPD, caused by iatrogenic immunodeficiency due to rheumatoid arthritis treatment, appeared to be less likely to occur. Due to the vaguely preserved lymph node structure and relatively small caliber of the CD30 it has been concluded that immunoreactive blastoid elements are more likely to be reactive immunoblasts than Hodgkin cells (Fig. 1.6).

Analysis of bone marrow biopsies demonstrated prominent eosinophilic granulocytes within the myeloid group and absence of CD30 lymphocytes. Due to the vaguely preserved lymph node structure and relatively small caliber of the CD30 it has been concluded that immunoreactive blastoid elements are more likely to be reactive immunoblasts than Hodgkin cells (Fig. 1.6). It is important to acknowledge that biopsies were obtained following administration of steroid therapy, which could have influenced the outcomes.

The patient rapidly recovered with disappearance of the skin rash, improvement of the liver function and overall decrease of the lymphocytosis. He was discharged from hospital after 10 days with supportive treatment of decreasing doses of methylprednisolone, pantoprazole, paracetamol and vitamin D substitution with calcium.

The day following his discharge, the patient consulted the Brussels University Hospital Emergency Department for relapse of acute fever (39,7° C) and thoracic and dorsal maculopapular erythema during the night. He presented with hypotension (100/52 mm Hg) and tachycardia (118 bpm). Cardiac monitoring and thoracic X-ray were normal. Abdominal echography suggested liver steatosis and splenomegaly. Laboratory data showed greatly disturbed liver tests (LDH 5508 U/L, AST 1417 U/L, ALT 2101 U/L), mild leukocytosis (10,100/mm³) with normal eosinophilic count (354/mm³, 3.5%), thrombocytopenia (52,000/mm³) and significant inflammation (CRP 53.5 mg/L). Only a few small submandibular lymph nodes were palpated. Viral serology was not tested.

The patient was admitted to the Department of Internal Medicine in Brussels, where again a dose of methylprednisolone (up to 48 mg/d or 0.6 mg/kg/day) was administered together with mometasone furoate 0.1% lipophilic cream on the skin lesions. Further investigations revealed significant hepatic cytolysis with liver steatosis, which evolved favorably under the corticosteroid treatment. The paroxysmal hyperglycemia secondary to the corticosteroid treatment was treated with insulin. Clinically neither arthritis nor synovitis was diagnosed.

The patient was discharged from hospital one week later. At home the corticosteroid dosing slowly decreased because of tremors, sleep disturbances and face swelling.

Six weeks later, liver function, leukocytosis, eosinophilic count and platelets had returned to normal values. Two months later the methylprednisolone treatment was stopped. No relapses occurred since. The patient attended two follow-up appointments, one a month later and another three months thereafter. Although further appointments were recommended, they did not occur. Laboratory analyses were conducted during these follow-ups, but imaging was not performed.

The most reasonable diagnosis was a relapse of the DRESS with reactive lymphadenopathy and hepatosplenomegaly despite treatment with high-dose methylprednisolone. The patient had a RegiSCAR score 7 out of 7 for DRESS during the relapse.

## Discussion

DRESS syndrome is a rare and live-threatening disease. Typical clinical presentations of the DRESS syndrome are fever, skin lesions and internal organ involvement [7].

Drugs reported to be a causative agent of DRESS syndrome include anti-convulsant, anti-bacterial and anti-tuberculosis agents, anti-retroviral drugs, anti-hepatitis C virus agents, antipyretics and analgesics, sulfonamides, proton-pump inhibitors, allopurinol and strontium ranelate. Severe cutaneous reactions are very rarely reported in clinical trials [6]. Some drug agents caused hypersensitivity reactions independent of the dose and the duration of the agent use, while others seem to correlate with the dose, duration and usage cycles [6, 11].

The pathogenesis of DRESS syndrome are still largely unknown, but there is evidence that drug hypersensitivity involves the reactivation of human herpesviruses (HHV-6) and the subsequent anti-viral immune response, as well as genetic predisposition, as reported by Wu et al. (2018) [12]. They investigated genetic factors associated with DRESS, with HLA-B*1301 being the most closely related allele. They concluded that EBV and HHV-6 detections may predict the prognosis of patients with DRESS. In their study, Cho et al. (2017), [7] also suggest that the reactivation of human herpesviruses and the ensuing anti-viral immune responses may contribute to the severity and prolonged course of this condition. Pathogenesis may include drug or metabolite accumulation due to altered enzymatic activity in some individuals; activation of drug-specific T lymphocytes in genetically predisposed persons; resulting in the clinical manifestations of DRESS syndrome. Conversely, viral reactivations may stem from direct drug or metabolite effects or a “cytokine storm” triggered by anti-drug immune responses; and these reactivations can induce robust antiviral responses, contributing to disease development.

Krishan et al. (2016) performed a multiple logistic regression model analyses on 48 sulfasalazine-induced DRESS case reports. They found that significant symptom parameters in the model involved hyper-eosinophilia, fever (> 38.5° C) and atypical lymphocytes [11]. Enlarged lymph ganglia are often a part of the clinical presentation of DRESS syndrome. In some cases, particularly following anticonvulsant drugs (lamotrigine, phenytoin, carbamazepine), the lymphadenopathy may include histopathological findings similar to lymphomas with CD30 positivity, in particular cutaneous CD30 + lymphoma [12–17]. Liver injury is one of the most common types of organ damage, found in 75–94% of patients [2, 3, 5, 7].

Lymphadenopathy is frequently observed in studies and takes part in diagnosing DRESS disease using scoring systems such as RegiSCAR, Japanese consensus group and criteria on Bocquet et al. (1996) (Table 1). However, the presence of lymphadenopathy introduces a diverse range of potential differential diagnoses with several malignant conditions, making histological analyses important for establishing an accurate diagnosis.

In our case, histological analysis of the lymph nodes could not differentiate between classical Hodgkin’s Disease and iatrogenic immunodeficiency/EBV-associated lymphoproliferative disorder (LPD), Hodgkin type. But the negative EBV in situ hybridization makes the presence of an EBV associated LPD, less likely. However, the relatively small caliber of the CD30 + immunoreactive blastoid cells in the lymph nodes suggests reactive immunoblasts rather than Hodgkin cells. Analysis of bone marrow biopsies revealed striking eosinophilic granulocytes in the myeloid group and absence of CD30 lymphocytes. However, it must be noted that biopsies were taken after administering the steroid therapy, which might have affected the results. Further investigations pointing to a sulfasalazine-induced DRESS syndrome, the EBV negativity and the morphologic aspect of the lymph node biopsies with predominance of T-cells were compatible with this diagnosis.

Managing DRESS syndrome mainly requires early removal of the causative agent and treatment with corticosteroids.

Our patient was initially discharged from the hospital after 10 days of treatment, but the next day, he had to be readmitted due to relapse of the symptoms. Interestingly, Krishan et al. (2016) conducted a meta-analysis of 48 cases of Sulfasalazine-induced DRESS syndrome, revealing that the average clinical treatment duration was approximately 2.27 weeks, significantly longer than our patient’s initial 10-day hospitalization period. They conclude that delayed and/or longer resolution time is the characteristic feature of DRESS, however, they also depict the general lack of research consensus on corticosteroids dosage and DRESS management [11].

A study by Cho et al. (2017) concludes that the duration of DRESS illness is usually more than 15 days [7]. Additionally, other studies suggest a prolonged 2-to-3-month use of corticosteroids with a gradual tapering [10, 18]. Shiohara and Mizukawa (2019) propose their DRESS scoring system, therapy length and dosage of corticosteroids depending on severity of patient symptoms [19].

Some patients might have complications, such as arthralgias, after several months, and should be followed up during several years [20]. For example, Hernández, Borrego, Soler, and Hernández (2013) followed up a Sulfasalazine DRESS patient within three years after discharge, confirming that patient was asymptomatic and receiving no treatment [21].

## Conclusion

We present a case of sulfasalazine-induced DRESS syndrome complicated by concurrent histologic features of CD30 + lymphoma. This case underscores the challenges in diagnosing and managing DRESS syndrome, which often has complications given by a diverse array of clinical and histopathological features. Notably, the histological analysis of skin demonstrated CD30 + immunoreactive blastoid cells, that together with clinical observations, results of CT-scans and FDG-PET CT-scan raised concerns for Hodgkin lymphoma, but histological results were later suggestive of reactive immunoblasts. The patient’s clinical course was marked by two hospital admissions, highlighting the need for prolonged patient monitoring in cases of DRESS syndrome. The duration of the illness and the variable response to corticosteroid treatment underscore the complexities in DRESS management, emphasizing the importance of individualized care and close follow-up for these patients. Further research is needed to elucidate the underlying mechanisms, diagnostics and optimal therapeutic strategies for DRESS treatment.

## Data Availability

No datasets were generated or analysed during the current study.

## Abbreviations

DRESS: Drug Reaction with Eosinophilia and Systemic Symptoms
EBV: Epstein-Barr Virus
LPD: Lymphoproliferative
CRP: C-Reactive Protein
CT: Computer Tomografie
FDG PET scan: Fluorodeoxyglucose Positron Emission Tomographie Scan
CD: Cluster of Differentiation
LDH: Lactatedehydrogenase
AST: Aspartate Aminotransferase
ALT: Alanine Transaminase
